# 4-Methyl-7,8,9,10-tetra­hydro­cyclo­hepta­[*b*]indol-6(5*H*)-one

**DOI:** 10.1107/S1600536809007429

**Published:** 2009-03-06

**Authors:** M. Sridharan, K. J. Rajendra Prasad, A. Thomas Gunaseelan, A. Thiruvalluvar, R. J. Butcher

**Affiliations:** a Department of Chemistry, Bharathiar University, Coimbatore 641 046, Tamilnadu, India; b PG Research Department of Physics, Rajah Serfoji Government College (Autonomous), Thanjavur 613 005, Tamilnadu, India; c Department of Chemistry, Howard University, 525 College Street NW, Washington, DC 20059, USA

## Abstract

In the title compound, C_14_H_15_NO, the seven-membered ring exhibits a slightly distorted twist-boat conformation. The pyrrole ring forms a dihedral angle of 1.44 (10)° with the fused benzene ring. N—H⋯O hydrogen bonds form a centrosymmetric dimer and weak C—H⋯π inter­actions are also found in the crystal structure.

## Related literature

For a related crystal structure, see: Sridharan *et al.* (2008 ▶).
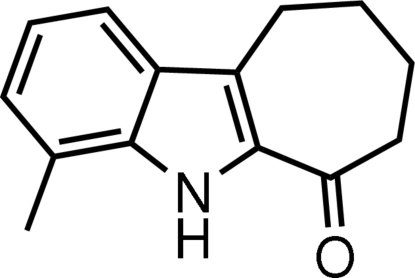


## Experimental

### 

#### Crystal data

C_14_H_15_NO
*M* *_r_* = 213.27
Monoclinic, 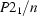

*a* = 9.6731 (4) Å
*b* = 10.0924 (5) Å
*c* = 11.8328 (6) Å
β = 103.397 (5)°
*V* = 1123.74 (10) Å^3^
*Z* = 4
Mo *K*α radiation
μ = 0.08 mm^−1^
*T* = 295 K
0.55 × 0.45 × 0.26 mm

#### Data collection

Oxford Diffraction Gemini R diffractometer
Absorption correction: multi-scan (*CrysAlis RED*; Oxford Diffraction, 2008 ▶) *T*_min_ = 0.936, *T*_max_ = 1.000 (expected range = 0.917–0.980)
9586 measured reflections
3772 independent reflections
2044 reflections with *I* > 2σ(*I*)
*R*_int_ = 0.025

#### Refinement

*R*[*F* ^2^ > 2σ(*F* ^2^)] = 0.077
*wR*(*F* ^2^) = 0.253
*S* = 1.04
3772 reflections
150 parameters
H atoms treated by a mixture of independent and constrained refinement
Δρ_max_ = 0.57 e Å^−3^
Δρ_min_ = −0.30 e Å^−3^

### 

Data collection: *CrysAlis CCD* (Oxford Diffraction, 2008 ▶); cell refinement: *CrysAlis RED* (Oxford Diffraction, 2008 ▶); data reduction: *CrysAlis RED*; program(s) used to solve structure: *SHELXS97* (Sheldrick, 2008 ▶); program(s) used to refine structure: *SHELXL97* (Sheldrick, 2008 ▶); molecular graphics: *ORTEP-3* (Farrugia, 1997 ▶); software used to prepare material for publication: *PLATON* (Spek, 2009 ▶).

## Supplementary Material

Crystal structure: contains datablocks global, I. DOI: 10.1107/S1600536809007429/wn2312sup1.cif

Structure factors: contains datablocks I. DOI: 10.1107/S1600536809007429/wn2312Isup2.hkl

Additional supplementary materials:  crystallographic information; 3D view; checkCIF report

## Figures and Tables

**Table 1:** Hydrogen-bond geometry (Å, °)

*D*—H⋯*A*	*D*—H	H⋯*A*	*D*⋯*A*	*D*—H⋯*A*
N5—H5⋯O6^i^	0.94 (3)	2.11 (3)	2.992 (2)	156.6 (19)
C10—H10*A*⋯*Cg*1^ii^	0.97	2.84	3.736 (2)	154
C14—H14*C*⋯*Cg*1^iii^	0.96	2.86	3.621 (2)	137
C8—H8*A*⋯*Cg*2^ii^	0.97	2.87	3.830 (3)	173

Symmetry codes: (i) 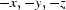; (ii) 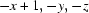; (iii) 
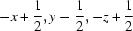
. *Cg*1 is the centroid of the pyrrole ring and *Cg*2 is the centroid of the benzene ring.

**Fig. 1.**
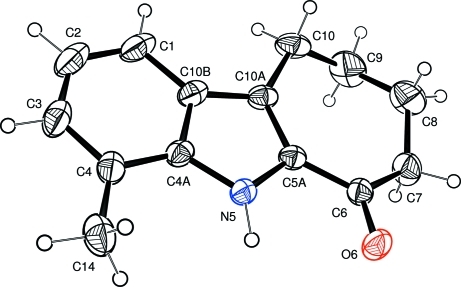
The molecular structure of the title compound, showing the atom-numbering scheme and displacement ellipsoids drawn at the 30% probability level. H atoms are shown as small spheres of arbitrary radius.

**Fig. 2.**
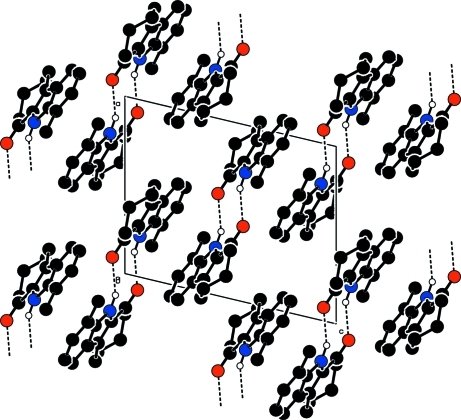
The packing of the title compound, viewed down the b axis. Dashed lines indicate hydrogen bonds. H atoms not involved in hydrogen bonding have been omitted.

## supplementary crystallographic information

### Comment

The title compound has been analysed as part of our crystallographic studies on cyclohept[b]indoles and their substituted analogues. Sridharan *et al.*(2008) have reported the X-ray crystal sructure of the related compound, 7,8,9,10-tetrahydro-2-methylcyclohepta[*b*]indol-6(5*H*)-one. In that paper, the seven-membered ring is stated to exhibit a slightly distorted envelope conformation.

In the title compound, C_14_H_15_NO (Fig. 1), the seven-membered ring exhibits a slightly distorted twist-boat conformation. The pyrrole ring forms a dihedral angle of 1.44 (10)° with the fused benzene ring.

N5—H5···O6(-*x*, -*y*, -*z*) hydrogen bonds form a centrosymmetric dimer. Furthermore, C10—H10A···π(1- *x*, -*y*, -*z*) and C14—H14C···π(1/2-*x*, -1/2+*y*, 1/2-*z*) interactions involving the pyrrole ring are present. Additionally, a C8—H8A···π(1-*x*, -*y*, -*z*) interaction involving the benzene ring are also found in the crystal structure.

### Experimental

A solution of 2-(2-(1-methylphenyl)hydrazono)cycloheptanone (0.230 g, 0.001 mol) in a mixture of acetic acid (20 ml) and conc. hydrochloric acid (5 ml) was refluxed on an oil bath pre-heated to 398–403 K for 4 h. The reaction was monitored by TLC. After the completion of the reaction, the contents were cooled and poured into ice water with stirring. The separated brown solid was filtered and purified by passing through a column of silica gel and eluting with a petroleum ether-ethyl acetate (95:5 v/v) mixture to yield the title compound (0.140 g, 66%). This product was recrystallized using ethanol.

### Refinement

H5 attached to  N5 was located in a difference Fourier map and refined isotropically; the final N—H distance was 0.94 (3) Å. The remaining H atoms were positioned geometrically and allowed to ride on their parent atoms, with C—H = 0.93, 0.96 and 0.97 Å for Csp^2^, methyl and methylene H atoms, respectively. *U*_iso_(H) = x*U*_eq_(C), where x = 1.5 for methyl H atoms and 1.2 for other C-bound H atoms.

### Crystal data

**Table:**

C_14_H_15_NO	*F*(000) = 456
*M**_r_* = 213.27	*D*_x_ = 1.261 Mg m^−^^3^
Monoclinic, *P*2_1_/*n*	Melting point: 412.5 K
Hall symbol: -P 2yn	Mo *K*α radiation, λ = 0.71073 Å
*a* = 9.6731 (4) Å	Cell parameters from 3217 reflections
*b* = 10.0924 (5) Å	θ = 4.7–32.7°
*c* = 11.8328 (6) Å	µ = 0.08 mm^−^^1^
β = 103.397 (5)°	*T* = 295 K
*V* = 1123.74 (10) Å^3^	Prism, colourless
*Z* = 4	0.55 × 0.45 × 0.26 mm

### Data collection

**Table:**

Oxford Diffraction Gemini R diffractometer	3772 independent reflections
Radiation source: fine-focus sealed tube	2044 reflections with *I* > 2σ(*I*)
graphite	*R*_int_ = 0.025
Detector resolution: 10.5081 pixels mm^-1^	θ_max_ = 32.8°, θ_min_ = 4.7°
φ and ω scans	*h* = −14→13
Absorption correction: multi-scan (*CrysAlis RED*; Oxford Diffraction, 2008)	*k* = −14→15
*T*_min_ = 0.937, *T*_max_ = 1.000	*l* = −17→15
9586 measured reflections	

### Refinement

**Table:**

Refinement on *F*^2^	Primary atom site location: structure-invariant direct methods
Least-squares matrix: full	Secondary atom site location: difference Fourier map
*R*[*F*^2^ > 2σ(*F*^2^)] = 0.077	Hydrogen site location: inferred from neighbouring sites
*wR*(*F*^2^) = 0.253	H atoms treated by a mixture of independent and constrained refinement
*S* = 1.04	*w* = 1/[σ^2^(*F*_o_^2^) + (0.1467*P*)^2^] where *P* = (*F*_o_^2^ + 2*F*_c_^2^)/3
3772 reflections	(Δ/σ)_max_ = 0.001
150 parameters	Δρ_max_ = 0.57 e Å^−^^3^
0 restraints	Δρ_min_ = −0.29 e Å^−^^3^

### Special details

**Table:**

Geometry. Bond distances, angles *etc*. have been calculated using the rounded fractional coordinates. All su's are estimated from the variances of the (full) variance-covariance matrix. The cell e.s.d.'s are taken into account in the estimation of distances, angles and torsion angles
Refinement. Refinement of *F*^2^ against ALL reflections. The weighted *R*-factor *wR* and goodness of fit *S* are based on *F*^2^, conventional *R*-factors *R* are based on *F*, with *F* set to zero for negative *F*^2^. The threshold expression of *F*^2^ > 2σ(*F*^2^) is used only for calculating *R*-factors(gt) *etc*. and is not relevant to the choice of reflections for refinement. *R*-factors based on *F*^2^ are statistically about twice as large as those based on *F*, and *R*- factors based on ALL data will be even larger.

### Fractional atomic coordinates and isotropic or equivalent isotropic displacement parameters (Å^2^)

**Table:**

	*x*	*y*	*z*	*U*_iso_*/*U*_eq_	
O6	0.07595 (15)	0.12098 (15)	−0.05930 (13)	0.0619 (5)	
N5	0.23136 (16)	−0.06788 (15)	0.07124 (12)	0.0407 (4)	
C1	0.5759 (2)	−0.1262 (3)	0.25005 (19)	0.0684 (9)	
C2	0.5665 (3)	−0.2535 (3)	0.2849 (2)	0.0765 (9)	
C3	0.4432 (3)	−0.3286 (3)	0.24770 (18)	0.0677 (9)	
C4	0.3234 (2)	−0.2780 (2)	0.17452 (17)	0.0523 (7)	
C4A	0.33242 (19)	−0.14568 (19)	0.14131 (14)	0.0430 (5)	
C5A	0.28716 (17)	0.05736 (17)	0.06406 (14)	0.0398 (5)	
C6	0.19212 (19)	0.15599 (19)	−0.00273 (15)	0.0445 (6)	
C7	0.2291 (3)	0.3012 (2)	0.0012 (2)	0.0648 (8)	
C8	0.3799 (3)	0.3459 (3)	0.0216 (3)	0.0855 (11)	
C9	0.4803 (3)	0.3090 (3)	0.1286 (3)	0.0892 (11)	
C10	0.5353 (2)	0.1680 (3)	0.1405 (2)	0.0624 (8)	
C10A	0.42772 (18)	0.05965 (19)	0.12696 (15)	0.0453 (6)	
C10B	0.45656 (19)	−0.0687 (2)	0.17618 (15)	0.0479 (6)	
C14	0.1923 (3)	−0.3579 (2)	0.1263 (2)	0.0715 (9)	
H1	0.65958	−0.07827	0.27466	0.0820*	
H2	0.64462	−0.29183	0.33504	0.0916*	
H3	0.44200	−0.41558	0.27329	0.0813*	
H5	0.134 (3)	−0.084 (2)	0.0449 (18)	0.054 (6)*	
H7A	0.17878	0.33949	−0.07201	0.0777*	
H7B	0.18946	0.34096	0.06134	0.0777*	
H8A	0.41736	0.31357	−0.04249	0.1026*	
H8B	0.37928	0.44191	0.01707	0.1026*	
H9A	0.43564	0.32612	0.19252	0.1070*	
H9B	0.56157	0.36766	0.13819	0.1070*	
H10A	0.59125	0.15438	0.08327	0.0749*	
H10B	0.59905	0.15903	0.21654	0.0749*	
H14A	0.18522	−0.37473	0.04526	0.1074*	
H14B	0.11011	−0.30959	0.13542	0.1074*	
H14C	0.19759	−0.44058	0.16719	0.1074*	

### Atomic displacement parameters (Å^2^)

**Table:**

	*U*^11^	*U*^22^	*U*^33^	*U*^12^	*U*^13^	*U*^23^
O6	0.0446 (8)	0.0549 (9)	0.0752 (10)	−0.0084 (6)	−0.0088 (7)	0.0151 (7)
N5	0.0350 (7)	0.0400 (8)	0.0453 (8)	−0.0003 (6)	0.0054 (6)	0.0036 (6)
C1	0.0434 (11)	0.102 (2)	0.0559 (12)	0.0226 (12)	0.0035 (9)	0.0005 (12)
C2	0.0640 (14)	0.107 (2)	0.0565 (13)	0.0439 (15)	0.0101 (10)	0.0127 (13)
C3	0.0818 (17)	0.0718 (15)	0.0551 (12)	0.0387 (13)	0.0272 (12)	0.0185 (11)
C4	0.0636 (12)	0.0531 (12)	0.0473 (10)	0.0179 (10)	0.0273 (9)	0.0090 (8)
C4A	0.0408 (9)	0.0520 (11)	0.0379 (8)	0.0101 (8)	0.0125 (7)	0.0001 (7)
C5A	0.0354 (8)	0.0431 (10)	0.0407 (8)	−0.0049 (7)	0.0083 (6)	−0.0033 (7)
C6	0.0427 (10)	0.0447 (10)	0.0442 (9)	−0.0046 (8)	0.0062 (7)	0.0041 (7)
C7	0.0683 (14)	0.0483 (12)	0.0718 (14)	−0.0129 (10)	0.0043 (11)	0.0063 (10)
C8	0.0817 (19)	0.0623 (16)	0.113 (2)	−0.0267 (14)	0.0236 (16)	0.0034 (15)
C9	0.0805 (18)	0.082 (2)	0.0953 (19)	−0.0445 (16)	0.0002 (15)	−0.0061 (15)
C10	0.0384 (10)	0.0861 (17)	0.0602 (12)	−0.0196 (10)	0.0065 (8)	−0.0131 (11)
C10A	0.0356 (9)	0.0608 (12)	0.0390 (8)	−0.0029 (8)	0.0074 (7)	−0.0079 (8)
C10B	0.0393 (9)	0.0648 (13)	0.0388 (9)	0.0101 (8)	0.0073 (7)	−0.0038 (8)
C14	0.095 (2)	0.0499 (12)	0.0785 (15)	0.0098 (12)	0.0380 (14)	0.0109 (11)

### Geometric parameters (Å, °)

**Table:**

O6—C6	1.220 (2)	C10—C10A	1.492 (3)
N5—C4A	1.373 (2)	C10A—C10B	1.421 (3)
N5—C5A	1.385 (2)	C1—H1	0.9300
N5—H5	0.94 (3)	C2—H2	0.9300
C1—C10B	1.402 (3)	C3—H3	0.9300
C1—C2	1.359 (4)	C7—H7A	0.9700
C2—C3	1.396 (4)	C7—H7B	0.9700
C3—C4	1.374 (3)	C8—H8A	0.9700
C4—C4A	1.401 (3)	C8—H8B	0.9700
C4—C14	1.500 (3)	C9—H9A	0.9700
C4A—C10B	1.409 (3)	C9—H9B	0.9700
C5A—C6	1.457 (3)	C10—H10A	0.9700
C5A—C10A	1.391 (2)	C10—H10B	0.9700
C6—C7	1.507 (3)	C14—H14A	0.9600
C7—C8	1.492 (4)	C14—H14B	0.9600
C8—C9	1.454 (5)	C14—H14C	0.9600
C9—C10	1.514 (4)		
			
O6···N5	2.684 (2)	H5···O6	2.41 (2)
O6···N5^i^	2.992 (2)	H5···C14	2.94 (2)
O6···H5	2.41 (2)	H5···H14B	2.5500
O6···H5^i^	2.11 (3)	H5···O6^i^	2.11 (3)
O6···H14B^i^	2.6300	H7A···H10B^vii^	2.4400
N5···O6	2.684 (2)	H7B···H9A	2.5300
N5···O6^i^	2.992 (2)	H7B···H14C^viii^	2.5300
N5···H14B	2.8800	H8A···H10A	2.5400
N5···H10A^ii^	2.9200	H8A···C2^ii^	2.9700
C3···C6^iii^	3.560 (3)	H8A···C3^ii^	3.0400
C6···C3^iv^	3.560 (3)	H9A···H7B	2.5300
C10A···C14^iv^	3.484 (3)	H9A···H14B^iv^	2.5800
C14···C10A^iii^	3.484 (3)	H10A···H8A	2.5400
C1···H10B	2.9200	H10A···H2^v^	2.5700
C2···H8A^ii^	2.9700	H10A···N5^ii^	2.9200
C3···H8A^ii^	3.0400	H10A···C4A^ii^	2.9200
C4A···H10A^ii^	2.9200	H10B···C1	2.9200
C10···H2^v^	3.0700	H10B···H1	2.5200
C10···H1	3.0400	H10B···H7A^ix^	2.4400
C10A···H14C^iv^	2.9600	H14B···N5	2.8800
C10B···H14C^iv^	2.9300	H14B···H5	2.5500
C14···H5	2.94 (2)	H14B···H9A^iii^	2.5800
H1···C10	3.0400	H14B···O6^i^	2.6300
H1···H10B	2.5200	H14C···H3	2.4200
H2···C10^vi^	3.0700	H14C···H7B^x^	2.5300
H2···H10A^vi^	2.5700	H14C···C10A^iii^	2.9600
H3···H14C	2.4200	H14C···C10B^iii^	2.9300
			
C4A—N5—C5A	109.03 (15)	C1—C2—H2	119.00
C4A—N5—H5	128.5 (13)	C3—C2—H2	119.00
C5A—N5—H5	121.0 (13)	C2—C3—H3	119.00
C2—C1—C10B	118.5 (2)	C4—C3—H3	119.00
C1—C2—C3	122.0 (2)	C6—C7—H7A	107.00
C2—C3—C4	122.1 (3)	C6—C7—H7B	107.00
C4A—C4—C14	120.54 (18)	C8—C7—H7A	107.00
C3—C4—C4A	115.7 (2)	C8—C7—H7B	107.00
C3—C4—C14	123.7 (2)	H7A—C7—H7B	107.00
N5—C4A—C4	129.27 (17)	C7—C8—H8A	107.00
N5—C4A—C10B	107.50 (16)	C7—C8—H8B	107.00
C4—C4A—C10B	123.21 (17)	C9—C8—H8A	107.00
N5—C5A—C6	116.80 (15)	C9—C8—H8B	107.00
C6—C5A—C10A	133.99 (17)	H8A—C8—H8B	107.00
N5—C5A—C10A	109.21 (15)	C8—C9—H9A	108.00
O6—C6—C7	118.74 (19)	C8—C9—H9B	108.00
C5A—C6—C7	122.15 (17)	C10—C9—H9A	108.00
O6—C6—C5A	119.02 (17)	C10—C9—H9B	108.00
C6—C7—C8	121.0 (2)	H9A—C9—H9B	107.00
C7—C8—C9	119.6 (3)	C9—C10—H10A	108.00
C8—C9—C10	118.1 (3)	C9—C10—H10B	108.00
C9—C10—C10A	117.22 (19)	C10A—C10—H10A	108.00
C5A—C10A—C10B	106.20 (16)	C10A—C10—H10B	108.00
C10—C10A—C10B	123.93 (17)	H10A—C10—H10B	107.00
C5A—C10A—C10	129.83 (18)	C4—C14—H14A	109.00
C4A—C10B—C10A	108.03 (16)	C4—C14—H14B	109.00
C1—C10B—C4A	118.49 (19)	C4—C14—H14C	110.00
C1—C10B—C10A	133.5 (2)	H14A—C14—H14B	109.00
C2—C1—H1	121.00	H14A—C14—H14C	109.00
C10B—C1—H1	121.00	H14B—C14—H14C	109.00
			
C5A—N5—C4A—C4	179.17 (18)	N5—C5A—C6—C7	167.99 (18)
C5A—N5—C4A—C10B	−1.88 (19)	C10A—C5A—C6—O6	172.63 (19)
C4A—N5—C5A—C6	−177.17 (15)	C10A—C5A—C6—C7	−11.0 (3)
C4A—N5—C5A—C10A	2.05 (19)	N5—C5A—C10A—C10	176.20 (19)
C10B—C1—C2—C3	1.2 (4)	N5—C5A—C10A—C10B	−1.36 (19)
C2—C1—C10B—C4A	−0.1 (3)	C6—C5A—C10A—C10	−4.8 (3)
C2—C1—C10B—C10A	177.7 (2)	C6—C5A—C10A—C10B	177.67 (18)
C1—C2—C3—C4	−0.7 (4)	O6—C6—C7—C8	−153.2 (2)
C2—C3—C4—C4A	−1.0 (3)	C5A—C6—C7—C8	30.4 (3)
C2—C3—C4—C14	175.8 (2)	C6—C7—C8—C9	−60.2 (4)
C3—C4—C4A—N5	−179.06 (19)	C7—C8—C9—C10	75.5 (4)
C3—C4—C4A—C10B	2.1 (3)	C8—C9—C10—C10A	−56.8 (3)
C14—C4—C4A—N5	4.0 (3)	C9—C10—C10A—C5A	27.5 (3)
C14—C4—C4A—C10B	−174.77 (19)	C9—C10—C10A—C10B	−155.4 (2)
N5—C4A—C10B—C1	179.35 (17)	C5A—C10A—C10B—C1	−177.8 (2)
N5—C4A—C10B—C10A	1.0 (2)	C5A—C10A—C10B—C4A	0.2 (2)
C4—C4A—C10B—C1	−1.6 (3)	C10—C10A—C10B—C1	4.5 (3)
C4—C4A—C10B—C10A	−179.94 (17)	C10—C10A—C10B—C4A	−177.54 (18)
N5—C5A—C6—O6	−8.4 (2)		

Symmetry codes: (i) −*x*, −*y*, −*z*; (ii) −*x*+1, −*y*, −*z*; (iii) −*x*+1/2, *y*−1/2, −*z*+1/2; (iv) −*x*+1/2, *y*+1/2, −*z*+1/2; (v) −*x*+3/2, *y*+1/2, −*z*+1/2; (vi) −*x*+3/2, *y*−1/2, −*z*+1/2; (vii) *x*−1/2, −*y*+1/2, *z*−1/2; (viii) *x*, *y*+1, *z*; (ix) *x*+1/2, −*y*+1/2, *z*+1/2; (x) *x*, *y*−1, *z*.

### Hydrogen-bond geometry (Å, °)

**Table:**

*D*—H···*A*	*D*—H	H···*A*	*D*···*A*	*D*—H···*A*
N5—H5···O6^i^	0.94 (3)	2.11 (3)	2.992 (2)	156.6 (19)
C10—H10A···Cg1^ii^	0.97	2.84	3.736 (2)	154
C14—H14C···Cg1^iii^	0.96	2.86	3.621 (2)	137
C8—H8A···Cg2^ii^	0.97	2.87	3.830 (3)	173

Symmetry codes: (i) −*x*, −*y*, −*z*; (ii) −*x*+1, −*y*, −*z*; (iii) −*x*+1/2, *y*−1/2, −*z*+1/2.
